# First steps towards assessing the evolutionary history and phylogeography of a widely distributed Neotropical grassland bird (Motacillidae: *Anthus correndera*)

**DOI:** 10.7717/peerj.5886

**Published:** 2018-11-21

**Authors:** Heraldo V. Norambuena, Paul Van Els, Carlos P. Muñoz-Ramírez, Pedro F. Victoriano

**Affiliations:** 1 Departamento de Zoología, Facultad de Ciencias Naturales y Oceanográficas, Universidad de Concepción, Concepción, Chile; 2 Centro de Estudios Agrarios y Ambientales, Valdivia, Chile; 3 Groningen Institute for Evolutionary Life Sciences, University of Groningen, Groningen, Netherlands; 4 Department of Biological Sciences and Museum of Natural Science, Louisiana State University, Baton Rouge, United States of America; 5 Facultad de Ciencias, Universidad Católica de la Santísima Concepción, Concepción, Chile; 6 Centro de Investigación en Biodiversidad y Ambientes Sustentables (CIBAS), Universidad Católica de la Santísima Concepción, Concepción, Chile

**Keywords:** South America, Andes, Pleistocene, Oscines, Lowlands, Highlands, Speciation

## Abstract

Grasslands in southern South America are extensive ecosystems which harbor a unique biodiversity; however, studies on the evolution of their taxa are scarce. Here we studied the phylogeography and population history of the Correndera Pipit (*Anthus correndera*), a grassland specialist bird with a large breeding distribution in southern South America, with the goals of investigating its phylogeographic history and relate it to the historical development of South American grasslands. The mitochondrial NADH dehydrogenase subunit II gene (ND2) was sequenced in 66 individuals from 19 localities and the intron 9 of the sex-linked gene for aconitase (ACOI9) was sequenced from a subset of those individuals, including all five subspecies of *A. correndera*, as well as the closely related *A. antarcticus*. Phylogenetic analysis revealed two distinct lineages within the complex: the first (A) corresponding to Andean subspecies *A. c. calcaratus* and *A. c. catamarcae* and the second (B) including birds traditionally assigned to *A. c. correndera*, *A. c. chilensis*, *A. c. grayi* and some individuals of *A. c. catamarcae*. *A. antarcticus* is nested within this second lineage. These results were also supported by evidence of niche divergence for variables associated with precipitation. The oldest split between clade A and B was estimated at *c.* 0.37 Mya, during the middle Pleistocene. Species distribution models for the present and the Last Glacial Maximum (LGM) suggest that grassland areas in southern South America remained relatively stable, in contrast to the general view of a reduction in grassland cover in South America since the LGM. Recent divergences and low phylogeographic structure (for lowland vs. highland geographic groups, intra-population genetic variance was greater than inter-groups; e.g., for ACOI9: 95.47% and ND2: 51.51% respectively), suggest widespread gene flow between lowland populations.

## Introduction

Geological processes and past climatic changes have been determining factors in generating complex patterns of genetic diversity (Hewitt, 1996). Both the spatial heterogeneity and the temporal variation of environmental conditions have influenced both intra- and interspecific diversification (Brown et al., 2014; Carnaval et al., 2009). As such, historical changes in habitat connectivity resulted in an increase in beta diversity, both at intra- and inter-specific level. The phylogeographic context of southern South America (central Andes to Patagonia) is complex, with different historical scenarios depending on latitude and habitat type. For example, in Patagonia a series of refugia have been described in glacial valleys, lowlands, and periglacial zones (e.g.,  Sérsic et al., 2011; Breitman et al., 2012; Cosacov et al., 2013). These areas maintained high levels of genetic diversity and worked as reservoirs for post-glacial colonization (Sérsic et al., 2011; Birks, 2015). Our knowledge of the historical processes driving diversity patterns in southern South America has increased in recent decades, mostly based on phylogeographic studies of organisms associated with temperate forest (Beheregaray, 2008; Sérsic et al., 2011). However, this has limited our understanding about the role of other important ecosystems, like grasslands, in generating biodiversity patterns and processes. This is a relevant issue given the dominance of grasslands in southern South America. Studying grassland specialist species will increase our understanding of the processes acting across different environments and their relative importance in causing regional patterns of biodiversity.

The study of diversification in Neotropical birds has been centered largely on the rich Amazonian and Andean forest biota, as illustrated by an abundance of recent phylogenetic and phylogeographic studies (e.g.,  Fjeldså, Bowie & Rahbek, 2012; Fernandes et al., 2015; Harvey & Brumfield, 2015). However, approximately 15% of South America is covered in various types of natural open lowland and montane grasslands (Eva et al., 2004). The Central Andes are considered a strong vicariant force for lowland-adapted taxa, isolating ancestral populations on both sides of the cordillera (Chapman, 1926; Brumfield & Capparella, 1996; Miller et al., 2008; Victoriano et al., 2015; Rivera et al., 2016). At the same time, the Andes are a source of diversification for highland taxa, particularly during glacial cycles (Weir, 2006; Weir, 2009; Sedano & Burns, 2010; Gutiérrez-Pinto et al., 2012; Valderrama et al., 2014; Beckman & Witt, 2015). This suggests that the diversification of certain widely distributed species inhabiting both montane environments and lowland grassland could be due to a set of processes including orogeny and changes in the distribution of habitats. Unlike in the Holarctic region, Neotropical grasslands are not distributed continuously and constitute a mosaic of different types of grasslands in the temperate (pampa), montane (highland moor), and (sub)tropical (closed, field, plains) zones (Suttie, Reynolds & Batello, 2005). The South American grasslands were more extensive during the Last Glacial Maximum (hereafter LGM, 20,000 years ago; Haffer, 1969) and probably during all glacial periods since *ca*. 1.5 Ma (Ruzzante et al., 2006) according to several lines of evidence including palynology (Salgado-Labouriau, 1991; Van Der Hammen, 1979), climate models (Markgraf, 1993) and fossil deposits (Webb, 1978) studied for the Amazonian region. This evidence suggests that many of the grasslands, and their fauna, were probably connected during the Pleistocene and became isolated only recently (Haffer, 1969), with the degree of connectivity likely varying with altitude and geographic location. In addition, genetic diversification can also be driven by differences in environmental conditions (Manel & Holderegger, 2013). For example, differences in extreme temperatures and precipitation have been shown to drive genetic divergence between populations of white-breasted Nuthatches from the sky islands (montane forest habitat islands in the USA) (Manthey & Moyle, 2015) and recent theory have emphasized the potential role of climatic differences in speciation (Nosil, 2012), especially in mountainous habitats where climatic conditions can shift abruptly across a smaller geographical space.

Phylogeographic studies on organisms inhabiting grasslands in Southern South America are scarce (e.g.,  Masello et al., 2011; Campagna et al., 2012a; Campagna et al., 2017), limiting our understanding about the potential historical scenarios that characterized these ecosystems as well as the potential processes shaping genetic diversity across taxa. Therefore, new information about the phylogeographic history of taxa that specialize in this type of habitat is crucial to understand the historical and geological processes that have shaped the biodiversity of this particular biome (Antonelli et al., 2010; Brumfield, 2012).

**Figure 1:**
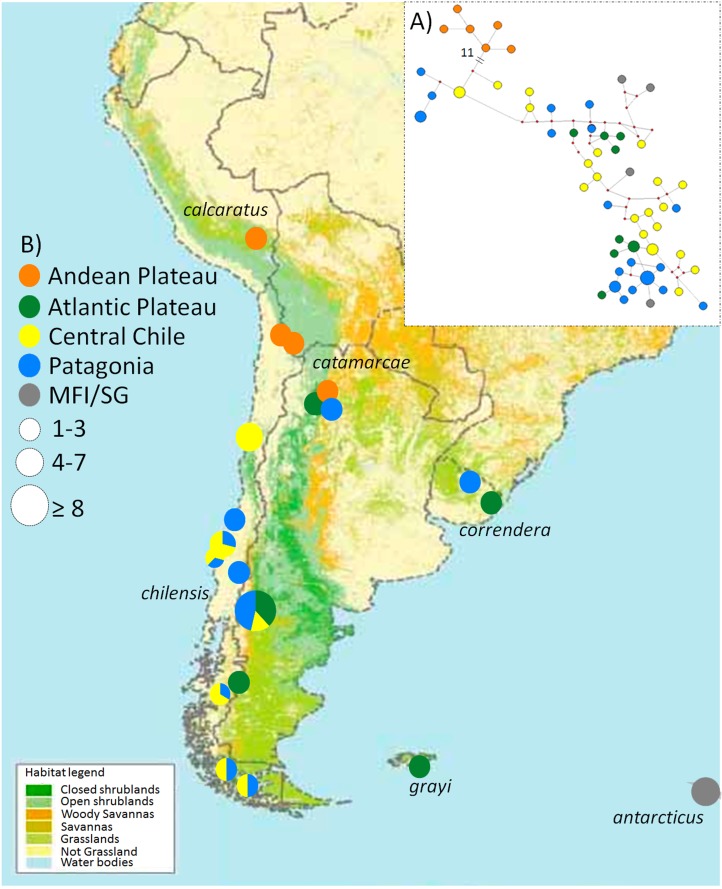
Map of the phylogeographic structure of *Anthus correndera* complex. Map of the phylogeographic structure of *Anthus correndera* complex showing: (A) the mtDNA ND2 haplotype network and the distribution of the five main haplogroups found in this study. (B) Pie charts represent the relative frequency of haplotypes within each locality and their sizes represent the sample size for that locality. At the bottom is the actual distribution of each grasslands and shrublands habitat in South America, data modified from Global Land Cover Characterization “International Geosphere-Biosphere Program” Dataset.

The Correndera Pipit (*Anthus correndera*) is a grassland bird, with a large breeding distribution in southern South America, present in a variety of grassland habitats including páramo and puna (both on the Andean Altiplano), pampas (Central and Eastern Argentina), Patagonian steppe, wetland pastures, and even vegetated dunes (Ridgely & Tudor, 1989). The Correndera Pipit is considered a polytypic species (Clements et al., 2016; Remsen Jr et al., 2016) with five recognized subspecies defined based on differences in plumage and geographic distribution or geographic isolation: *A. c. calcaratus* from Junín, Cuzco and Puno (Peru), *A. c. catamarcae* from northern Chile (highlands east of Antofagasta), southeastern Peru, southern Bolivia and northwestern Argentina (Catamarca), *A. c. correndera* from southern Paraguay, northeast and eastern Argentina, Uruguay and southeastern Brazil (Rio Grande do Sul), *A. c. chilensis* from Chile (southern Atacama to Tierra del Fuego), and *A. c. grayi* restricted to the Malvinas/Falkland Islands hereafter MFI (Fig. 1; Tyler, 2004; eBird, 2016). Most subspecies are resident (Tyler, 2004), but some Patagonian populations of *A. c. chilensis* migrate north during the austral winter (Norambuena et al., 2017).

Previous studies on the systematics of *A. correndera* reported that this species is phylogenetically close to *A. antarcticus*, and both are part of a clade that also includes other species from South America (Voelker, 1999). This work, based on the mitochondrial gene cytochrome-b, also suggests that the nominate and *catamarcae* are paraphyletic, with *correndera* being sister to *A. antarcticus* and *catamarcae* sister to this clade (Voelker, 1999). In addition, a phylogenetic study based on the mitochondrial gene COI, which included some samples from Patagonian subspecies (i.e., *A. c. grayi* and *A. c. chilensis*), noted that genetic differentiation between continental (*A. c. chilensis*) and island populations (*A. c. grayi*) was negligible. This could be explained by different levels of migration, differences in the effective population size, or by multiple colonization events from the continent to the MFI islands (mainland-island flow; Campagna et al., 2012b). Although this latter study suggests interesting phylogeographic processes and phylogenetic arrangements, a larger geographic sample and population-level analyses are needed. For instance, previous studies did not include samples from *A. antarcticus*, a key taxon in this species complex that is endemic to South Georgia and is the only passerine present in these islands. Although *A. antarcticus* is phenotypically clearly different from *A. correndera*, with a streaked, dark appearance and bigger size (Tyler, 2004), it has been suggested that this taxon could have differentiated after South Georgia was colonized by specimens of *A. correndera* (Tyler, 2004). However, recent information based on a multilocus analysis, suggests that *A. correndera* is a species complex, with *A. c. catamarcae* sister to *A. c. calcaratus*, *A. c. correndera* sister to *A. c. chilensis* and *A. c. grayi* sister to the species *A. antarcticus,* which is nested within this complex (Van Els & Norambuena, 2018).

Considering the complex history and geography of the habitat of *A. correndera* in southern South America and the uncharacterized evolutionary relationships within the species, the aim of this study is to evaluate the phylogeographic structure of *A. correndera* across most of its distributional range in order to test the following hypothesis predictions: (i) The degree of phylogeographic differentiation across the distribution of the species is not related to current morphology-based subspecific designations; (ii) the genetic structure is associated with environmental differences as represented by climatic variables; and (iii) the demographic history of lineages is related to the development of different types of grasslands across the South American landscape.

## Material and Methods

### Sampling

We sampled individuals from each subspecies in the Correndera Pipit complex, as well as from the close relative *A. antarcticus*. Our sampling included individuals of *A. c. calcaratus* (*N* = 2), *A. c. grayi* (*N* = 1), *A. c. correndera* (*N* = 4), *A. c. chilensis* (*N* = 43), *A. c. catamarcae* (*N* = 12), and *A. antarcticus* (*N* = 4), and covered most of the complex’s distribution (Fig. 1; see Table S1). We captured Chilean populations of *A. c. chilensis* and the *A. c. catamarcae* subspecies in the field using mist-nets, and each individual was measured and photographed. For genetic analysis, we collected blood samples by venipuncture of the brachial vein. We obtained a Chilean collecting permit from Servicio Agrícola y Ganadero (SAG-Chile) No. 7285/2015. Genetic samples from *A. antarcticus, A. c. calcaratus, A. c. grayi*, and *A. c. correndera* were obtained from museum tissues and skins (Table S1).

### DNA extraction, amplification and sequencing

Genomic DNA was extracted from samples following the protocol of Fetzner (1999) using the QIAGEN DNAeasy kit. We amplified the mitochondrial gene NADH dehydrogenase subunit II (ND2) for all the samples with the primers L5216 and H6313 following the protocol described in Sorenson et al. (1999), and the intron 9 of the sex-linked gene for aconitase (ACOI9) with the primers ACO1-I9F and ACO1-I9R following  Kimball et al. (2009) for a subset of samples that included representatives of all subspecies of *A. correndera* (Table S1). PCR products were sequenced in both directions through automatic sequencing using Macrogen’s ABI3730XL (Seoul, South Korea). Sequences were edited using Codon Code Aligner v. 3.0.3 (CodonCode Corporation, www.codoncode.com), and translated into amino acids to corroborate the absence of stop codons. To detect and interpret insertions and deletions in the nucDNA, we used the program Indelligent (Dmitriev & Rakitov, 2008). We phased ACOI9 sequences in DnaSP v.5 (Librado & Rozas, 2009) using the algorithm provided by PHASE (Stephens & Donnelly, 2003a; Stephens & Donnelly, 2003b), with an ambiguity cutoff of >0.7. When sequences from nuclear markers presented heterozygous sites, haplotypes were inferred using the coalescent-based Bayesian method implemented in Phase 2.1 (Stephens & Donnelly, 2003b; Stephens & Scheet, 2005; Stephens, Smith & Donnelly, 2001). A probability threshold was first established at 0.9, but as some haplotypes were resolved, we then lowered the threshold to 0.6 following Garrick, Sunnucks & Dyer (2010), who suggested that this value increases the number of resolved haplotypes with almost no increase in false positives. Sequence alignments were conducted in MUSCLE (Edgar, 2004) producing a final alignment length of 1,038 bp for 63 samples of ND2 and 1,026 bp for 19 samples of ACOI9. A saturation test was conducted in DAMBE v. 5.2 (Xia, 2013) to evaluate the utility of sequences for phylogenetic analyses. The proportion of invariable sites, a key parameter for the saturation test, was obtained with jModeltest 2 (Darriba et al., 2012). All sequences have been deposited in GenBank (accession numbers MH781103 –MH781135, Table S1).

### Phylogenetic analysis and divergence times

We used both Bayesian inference (BI) and maximum likelihood (ML) approaches for phylogenetic reconstruction. We reconstructed a tree using only the ND2 gene and a species tree using both ND2 and ACOI9 for a subset of individuals. We identified the best-fit nucleotide substitution model for each gene using jModeltest 2 (Darriba et al., 2012) which indicated HKY+ Γ as the best-fit model for ND2 and also for ACOI9 under the Akaike information criterion. BI analyses were conducted using MrBayes 3.2.1 (Ronquist & Huelsenbeck, 2003) with the ND2 sequences, by means of two runs with four chains each. We performed two different runs; one using the HKY+ Γ evolutionary model for the aligned matrix of ND2 and the other using an evolution model partitioning each position of the codons in the ND2 matrix. We ran all analyses for 100 million generations and we sampled every 1,000 steps; the first 25% of the data was discarded as burn-in. The convergence of MCMC analysis was examined visually in Tracer v1.6 (Rambaut & Drummond, 2009). We then compared log-likelihood of each matrix model by Bayes Factor for model selection. We chose the partitioning model because it returned a score 81 log-likelihood units greater than the HKY+ Γ evolutionary model.

ML analyses were conducted in RAxML v8 (Stamatakis, 2014) using the multiple inference strategy. We ran 1,000 independent inferences and 1,000 bootstrap replicates with the same nucleotide substitution model settings as for the Bayesian analysis. Bootstrap support values were passed to the tree with the highest likelihood among the 1,000 independent tree inferences.

We also inferred a haplotype network by using the “median joining network” algorithm in Network 4.610 (Bandelt, Forster & Röhl, 1999), which is based on the sum of weighted differences (i.e., Hamming distance) between sequences. Ambiguities within the network were solved according to the criteria of Crandall & Templeton (1993).

The time of divergence of the most recent common ancestor (MRCA) of the main clades of *A. correndera* was estimated with BEAST v. 1.8.4 (Drummond et al., 2012), taking advantage of the BEAGLE library (Ayres et al., 2012). The Markov Chain Monte Carlo (MCMC) method within a Bayesian framework (BMCMC) was used to estimate the posterior probability of phylogenetic trees using the model HKG + Γ for the ND2 matrix. One hundred million trees were generated, sampling every 1,000 trees to assure that successive samples were independent. In order to estimate the timing of diversification in relation to Pleistocene glaciations we determined divergence times under four molecular clocks: strict, uncorrelated relaxed lognormal, random local and fixed local, and tested for the best-fit model by Bayes Factor. We chose the strict clock because it returned a score 20 log-likelihood units greater than the other clocks. To produce a time-calibrated tree we used a substitution rate prior of 0.0125 substitutions/site/year (2.5% divergence per million years; (Smith & Klicka, 2010) and a ‘calibrated Yule model’ for tree prior, fixing the node leading to *A. spraguei* at 4.55 Mya, which is the mean estimated age of a Pliocene fossil pipit from Kansas (Emslie, 2007). For this model, we used 1/x distributions for clock rate priors. In all analyses the first 25% of the trees were discarded as burn-in. The convergence of BMCMC analyses was examined visually in the program Tracer v1.6 (Rambaut & Drummond, 2009) to check for stationarity and effective sample sizes (ESS) above 200. We also recovered a species tree in *BEAST, a component of BEAST v. 2.3.2 ( Drummond & Rambaut, 2007) by using the individuals for which both ND2 and ACOI9 sequences were available (see Table S1). We used the same nucleotide substitution and model settings as used for the BEAST analysis, and ND2 and ACO1 as two independent loci.

For all the analyses we used sequences from *A.  nattereri*, *A. bogotensis meridae*, *A. bogotensis bogotensis*, *A. bogotensis immaculatus*, *A. bogotensis shiptoni*, *A. hellmayri brasilianus*, *A. hellmayri dabbenei*, *A. hellmayri hellmayri*, *A. chacoensis*, *A. lutescens peruvianus*, *A. spraguei*, *A. cinnamomeus*, *A. gustavi*, *A. rubescens* and *A. rufulus* as out-group (Table S1).

### Population genetic analyses and historical demography

The number of polymorphic sites (S), haplotype diversity (H) and nucleotide diversity (*π*) were calculated in DnaSP v.5 (Librado & Rozas, 2009). To evaluate the occurrence of recent population expansion we calculated Tajima’s *D* test (Tajima, 1989) and Fu’s *Fs* test (Fu, 1997) in Arlequin 3.5 (Excoffier & Lischer, 2010). To estimate gene flow between populations, we used the Migrate-n version 3.6.4 (Beerli, 2006; Beerli & Felsenstein, 2001) under a Bayesian coalescent framework (Kingman, 1982). In order to obtain the posterior distribution of the number of immigrants per generation (Nm), we analyzed a pair-wise comparison of populations based on mtDNA (ND2): clade A versus clade B. A starting UPGMA tree was used and the initial theta (0–0.1) and M (0–1,000) values were uniform. Static heating was applied to all four independent chains using the temperature settings of 1.0, 1.5, 3.0 and 1,000,000.0. A total of 10,000,000 steps were run and recorded every 1,000 generations, from which 10,000 were discarded as the burn-in. Stationarity was assessed by examining the effective sample size (ESS) and distribution of each parameter in Tracer v1.6 (Rambaut & Drummond, 2009).

Distinct hierarchical analyses of the distribution of genetic diversity of *A. correndera*, for two genes (ND2 and ACOI9), were conducted in the form of analysis of molecular variance (Amova) using Arlequin 3.5 (Excoffier & Lischer, 2010). Hierarchical levels were defined on the basis of taxonomic groups and phylogeny results. Amova groups were constructed both by taxonomic groups, and by lowland and highland individuals.

We conducted Bayesian Skyline Plot (BSP) analyses in BEAST v. 1.8.4 (Drummond et al., 2012) to estimate changes in effective population size since the most recent common ancestor (TMRCA), using the same substitution model as described above for the Bayesian tree analysis (Darriba et al., 2012). A strict clock model was set with the same clock rate as above, and with Coalescent: Bayesian Skyline selected. MCMC chain was run for 50 million generations and sampled every 1,000 generations. The first 25% of samples was discarded as burn-in. Tracer v1.6 (Rambaut & Drummond, 2009) was used to both visualize the log files resulting from the analysis and to generate the BSP.

### Ecological niche modelling (ENM)

Species distribution models (SDM) for *A. correndera* were generated using MAXENT v3.3.3k (Phillips et al., 2006) to investigate the relationship between phylogeographic structure and habitat discontinuities. Occurrence points were obtained from eBird (http://ebird.org/content/ebird/), from the literature (e.g., Ridgely & Tudor, 1989; Jaramillo, 2003), and from our own sample collections for a total of 6,327 records that were later reduced to 439 by curation and filtering (Fig. S1). To reduce spatial autocorrelation that usually results from sampling areas with a high density of locality points (clusters of points), we spatially filtered locality data to allow a minimum distance of 10 kilometers between any two points. We used 19 bio-climatically informative variables (WORLDCLIM v1.4; Hijmans et al., 2005) to represent present-day distributions and past (LGM) distributions (PMIP2- CCSM; Braconnot et al., 2007), both models with a resolution of 2.5 arc-minutes. Although MAXENT is generally robust to modelling with highly correlated variables (e.g., including all bioclimatic layers), we finally removed highly correlated variables using Pearson’s r correlation test until no pairwise correlation coefficient was greater than 0.8, to allow for better interpretations of the influence of variables on the SDMs. The variables kept were bio1, bio2, bio3, bio7, bio12, bio14, bio15, bio18, and bio19 (see details in Table S2). To avoid model overfitting (developing a model too specific for this set of data), we restricted the area for training the model by creating a bias file for background selection. Specifically, we created polygons with a buffer area of 100 km around the distribution of each species/lineage using the ‘Sample by buffered local adaptive convex-hull’ tool available in the SDMtoolbox v1.1c (Brown, 2014). This generates a bias file in *.asc* format which can be loaded into MAXENT in the advanced settings to sample background points. The R-package ENMeval (Muscarella et al., 2014) was used to evaluate the best parameter settings (e.g., feature classes and regularization multipliers) to be used in the ecological niche modeling procedure. Accordingly, the model was run using the following settings: maximum number of background points = 10,000; replicates = 10; and replicated run type = Crossvalidate. Based on the ENMeval results, we selected only threshold features and set the regularization multiplier to 1.5. All other settings were kept with default values. The performance of our ENM was evaluated with the Area under the Curve (AUC) provided by MAXENT, and the use of the threshold-dependent True Skill Statistics index (TSS) following Allouche, Tsoar & Kadmon (2006). To build distribution maps, resulting suitability maps had to be transformed to binary distribution maps. We tested the performance of two thresholds with the TSS test, the Minimum training presence logistic threshold (MTP) and the 10 percent training presence logistic threshold (10TP). The MTP threshold is the lowest suitability value that allows all localities to be included in the distribution map, whereas the 10TP threshold is the suitability value that allows the 90% of the localities to be included in the distribution map. The 10TP threshold can be useful for relatively inaccurate datasets as it allows for some level of uncertainty in the occurrence data set. Once we selected a threshold, we obtained stability maps by intersecting past and present species distributions, keeping only the overlapping regions as stable areas.

Principal Component Analyses (PCA) and a background test of niche divergence were conducted to investigate whether subspecies exhibit differences in climatic space. For each locality in the rarefied coordinate dataset (see the SDM section above), we collected the climatic data values from the same bioclimatic layers used in the SDM after highly correlated bio-climatic layers were removed. Statistical significance between groups was assessed using a PERMANOVA test. For the background test we followed McCormack, Zellmer & Knowles (2010). The background test compares niche divergence (differences in climate space) to a null model of background environmental differences and offers a powerful approach to test niche divergence while accounting for spatial autocorrelation. We used this test to evaluate whether there was evidence of niche divergence between the two main clades (A and B) resulting from the genealogical analysis. For a detailed description of this test see Supplementary material. All PCA analyses, statistical associated tests, and the background test were conducted in R (R Development Core Team, 2013).

## Results

### Phylogeographic structure and divergence times

**Figure 2:**
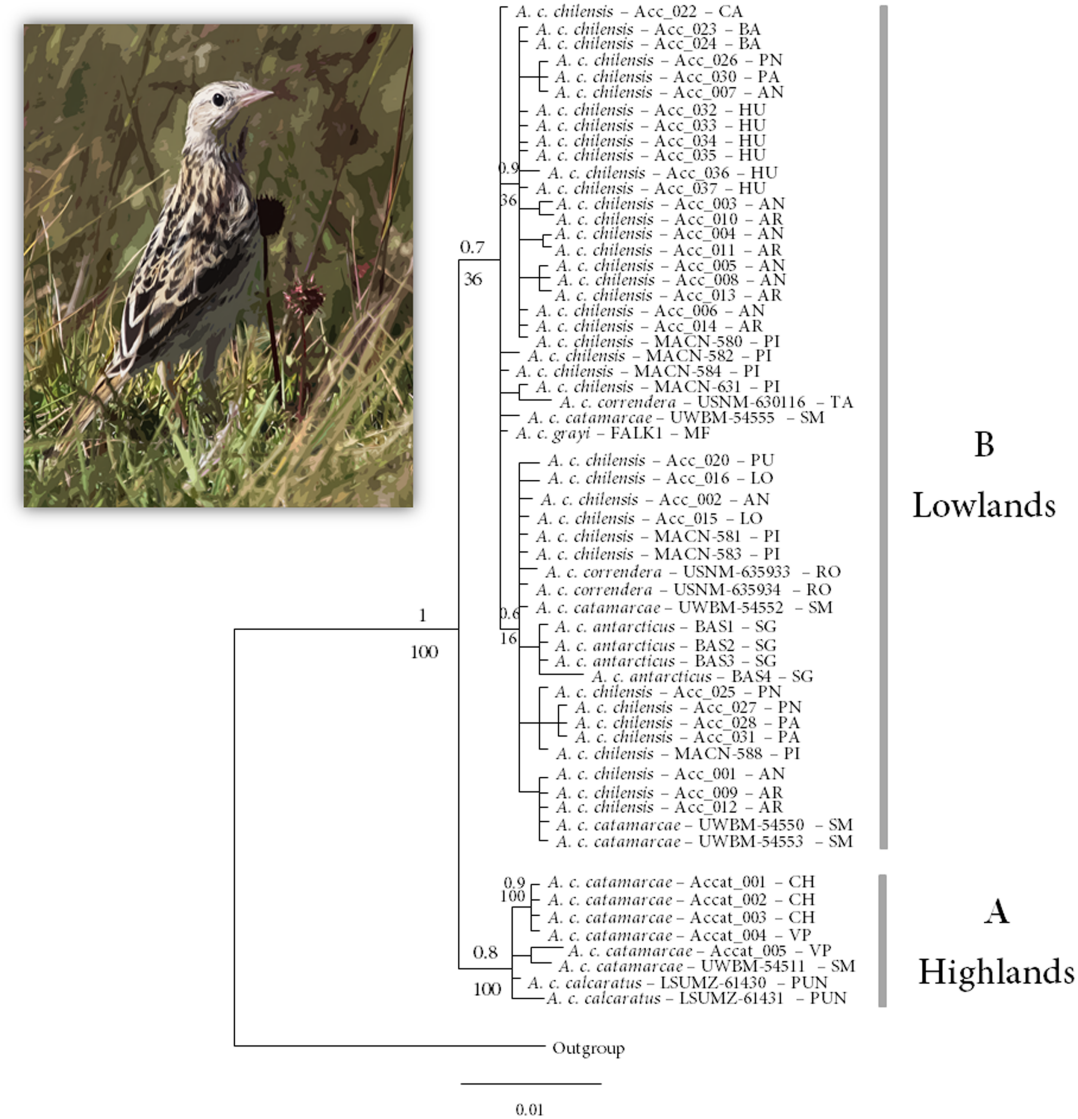
Bayesian and Maximum Likelihood tree representing the relationship within the *Anthus correndera* complex. Bayesian and Maximum Likelihood tree representing the relationship within the *Anthus  correndera* complex using mtDNA ND2 sequences. The values above the nodes correspond to the Posterior Probability values and under the nodes to Bootstrap values. Photo of *Anthus correndera* from Heraldo V. Norambuena.

Sequences 917 bp in length for the ND2 locus and 1,019 bp for ACOI9 were obtained, and each sequence showed low saturation (*p* ≤ 0.001). Twenty-one haplotypes were identified for the ND2 sequences, defined by 19 polymorphic sites (6 singletons, 13 parsimony informative sites). The ML and BI trees based on ND2 sequences showed similar topologies (Fig. 2). Both trees inferred the Correndera Pipit complex to be paraphyletic and composed of two well-supported clades (Clades A and B; posterior probability of 1.0 and ML bootstrap support of 100). Clade A included individuals that belong to the Andean Altiplano or Highlands subspecies *A. c. calcaratus* and *A. c. catamarcae* (Fig. 2), with a lack of internal structure in relation to subspecies monophyly (i.e., subspecies were not reciprocally monophyletic). Clade B included individuals from a wide lowland continental distribution ranging from Uruguay and Argentina to Chile and MFI and South Georgia. This clade consisted of birds traditionally assigned to *A. c. correndera*, *A. c. chilensis*, *A. c. grayi* and *A. antarcticus,* and also included some *A. c. catamarcae* individuals (Fig. 2). The species tree analysis for ND2 and ACOI9 sequences were broadly concordant with the mitochondrial tree, recovering two main clades with poor nodal support within each clade (Fig. S2).

The ND2 haplotype network revealed the same two major haplogroups that were recovered from the Bayesian phylogenetic and species tree reconstructions (Fig. 1A); most of the shared ND2 haplotypes among localities occur in the lowland range, specifically within a Central Chile and Patagonian distribution (Table S3). According to the molecular clock calibration, the older divergence within *A. correndera* (the split that originated clades A and B) dated to the middle Pleistocene, around *c.* 0.37 Mya (0.46–0.19 Mya; 95% HPD; Fig. S3).

### Population genetic analyses and historical demography

Overall haplotype diversity for the ND2 gene was 0.920 ±  0.019 and overall nucleotide diversity was 0.00561. Considering the absence of reciprocal monophyly associated with most of the subspecies, we tested for genetic differences between the clades retrieved by phylogenetic analysis (i.e., clade A vs. clade B). Clade B had the highest haplotype and nucleotide diversity, while clade A had the lowest values (Table 1). Sample sizes differ considerably between these groups and should be thus considered with caution.

**Table 1:** Genetic diversity statistics, tests for neutrality, and demographic expansion of mtDNA ND2 sequences for the clades retrieved by phylogenetic analysis in the *Anthus correndera* complex.

Clade/area	Taxa	*N*	*H*	Hd	Π	Tajima’ *D*	Fu’s *Fs*
A/highlands	*calcaratus* + *catamarcae*	8	5	0.786	0.00225	−0.63262(n.s.)	−1.152(n.s.)
B/lowlands	*correndera* + *chilensis* + *grayi* + *antarcticus*	50	19	0.916	0.00332	−1.23599(n.s.)	−11.527^*^

**Notes.**

*N* number of sequences *H* number of haplotypes Hd haplotype diversity *π* nucleotide diversity

* *p* < 0.05.

Tajima’s D values did not indicate evidence of population expansion either overall or within either clade. Fu’s test detected significant population expansion for clade B (Table 1). For the ND2-based AMOVA, most of the values were significant (*p* < 0.001). The largest fraction of the observed genetic variation was reached when samples were grouped according to taxonomy within populations with 63.98% (*P* < 0.001) for the highlands vs. lowlands grouping, and the results showed similar values among (48.49%) and within populations (51.51%) (Table 2). For the ACOI9-based AMOVA, there was more genetic variation within populations, with 95.47% (*P* < 0.001) for the highlands vs. lowlands grouping, and 81.64% (*P* < 0.001) for the taxonomy-based grouping (Table 2).

Despite the phylogenetic tree showing almost complete reciprocal monophyly for highlands–lowlands, our migration analysis indicated bidirectional gene flow (ΘM), with more gene flow from lowlands to highlands (112.5 vs. 2.5). The BSP recovered signs of population expansion in lowlands (Fig. S4).

### Species distribution models, niche differentiation, and genetic structure

**Figure 3:**
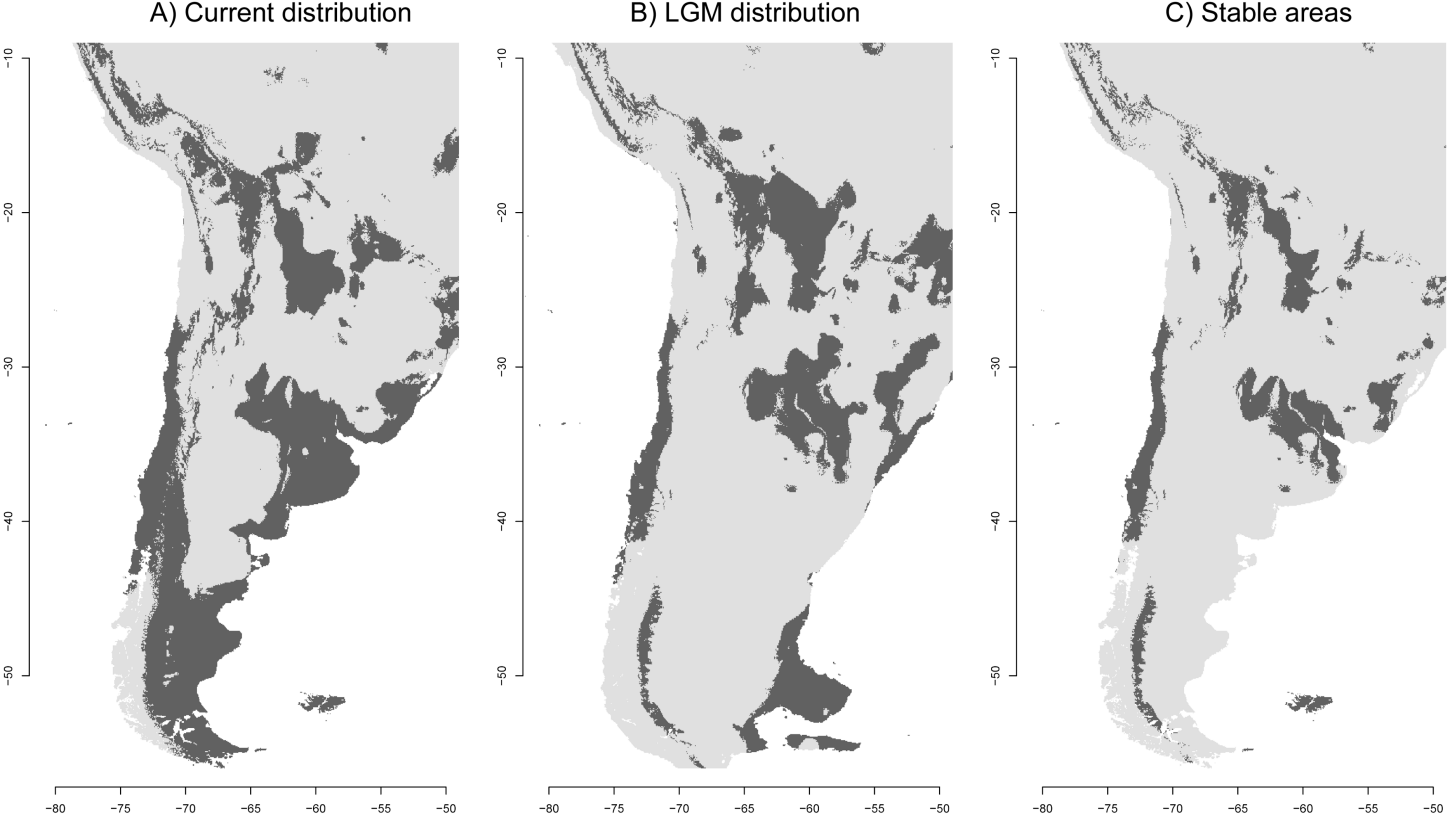
Ecological niche models of the *Anthus correndera* complex. (A) Current distribution. (B) LGM distribution. (C) Stable areas. Dark grey color indicates conditions typical of those where the species is found.

Present-day SDM predicted the current distribution of *A. correndera* fairly well (mean area under the curve, AUC = 0.764), except for some areas in eastern Brazil and in southern Andean Ecuador, where the species is not currently present (Fig. 3A). The TSS test found that the 10th percentile training logistic presence (10TP) threshold produced a better representation of the distribution of the species than the minimum training presence logistic threshold (TSS = 0.322, SD = 0.046 and TSS = 0.006, SD = 0.012, respectively). Accordingly, we use the distribution map obtained with the 10TP threshold to report our results and discussions.

**Table 2:** AMOVA results for ND2 and ACOI9 sequences. Toponymic for each group corresponds to taxonomy or phylogeny results.

Marker	Group level	Source of variation	*d*.*f*.	Sum of squares	Variance components	Percentage of variation (%)	Fixation index	*P*-value
ND2	Taxonomic	Among groups	5	53.36	133.74	36.02	0.36	<0.001
		Among populations within groups	7	192.31	1.65	8.69	0.08	0.11
		Within populations	53	125.92	237.58	63.98	0.64	<0.001
	Lowland vs. highland	Among groups	1	35.39	237.61	48.49	0.48	<0.001
		Among populations within groups	2	170.91	3.88	19.37	0.19	<0.001
		Within populations	57	143.89	252.44	51.51	0.52	<0.001
ACOI9	Taxonomic	Among groups	5	705.18	19.65	18.36	0.18	<0.001
		Among populations within groups	6	842.63	23.06	21.79	0.21	0.10
		Within populations	12	1.048.05	87.34	81.64	0.82	<0.001
	Lowland vs. highland	Among groups	1	140.24	4.85	4.53	0.04	0.99
		Among populations within groups	2	177.95	−2.69	−2.64	−0.02	0.60
		Within populations	16	1.629.14	102.20	95.47	0.95	<0.001

As expected, the current SDM shows a discontinuous distribution for the species in agreement with the discontinuous nature of the grasslands. The isolation of this northern area can be better seen in Fig. 3C, which suggests that a more stable area in the Altiplano remains disconnected from other areas further south. The distribution during the LGM reveals isolated populations in central Chile, a large area between northern Argentina and eastern Brazil, and in the Andes where some areas were reduced and others increased to the east (Fig. 3B). Interestingly, the Atlantic Plateau in southeastern Argentina appears connected with suitable grassland habitat to the MFI. During the LGM, South Georgia was larger in extent but did not have a suitable habitat for *A. correndera* (Fig. 3B). During the LGM, connectivity between Andes and lowlands was probably greater in the area of north-central Argentina (Fig. 3B). A striking result of comparing past and present distributions for *A. correndera* is that the total surface area seems to remain relatively constant, varying from ∼2,492,790 km^2^ during the LGM to ∼2,700,145 km^2^ in the present day, a growth of 8% in contrast to the historical decrease since the LGM that has been suggested previously for South American grasslands. Some stable areas (∼1,252,726 km^2^) were detected on the Andean Altiplano, in central Chile, northeastern Argentina, Patagonia and MFI (Fig. 3C).

**Figure 4:**
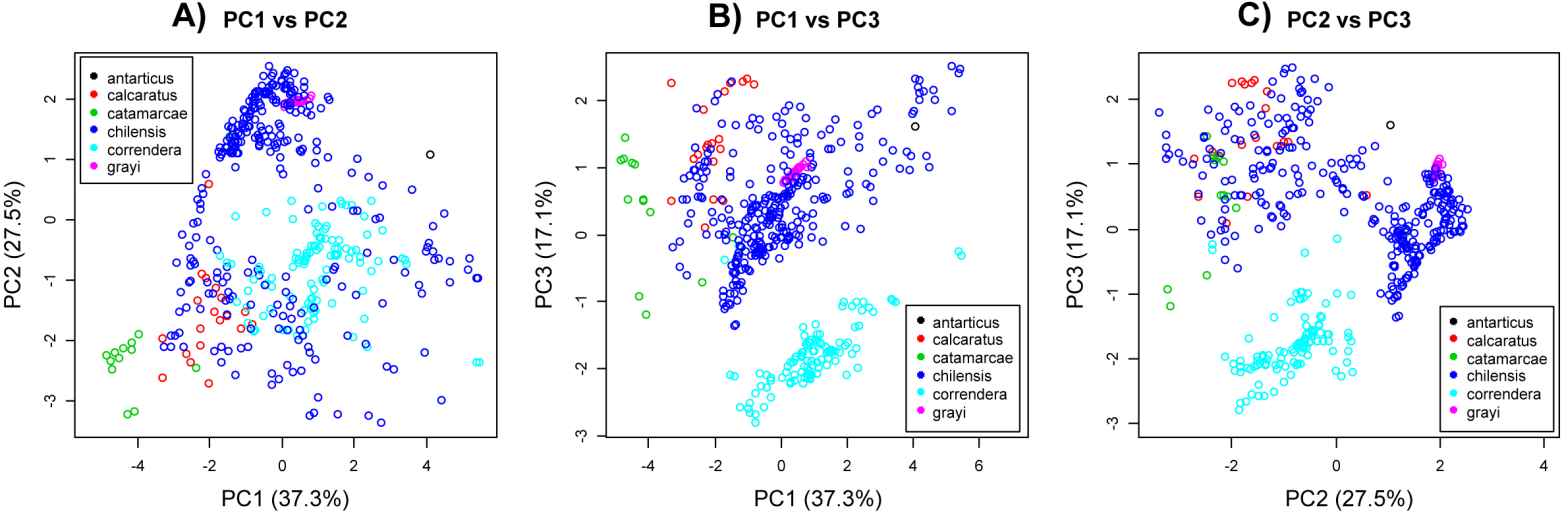
Principal component analysis. (A) PC2 vs. PC2. (B) PC1 vs. PC3. (C) PC2 vs. PC3. Principal component analysis of environmental variables associated with coordinate data for *Anthus correndera*. Percentage of variation explained by each PC axis is given within parenthesis. Colors correspond to the different subspecies of *A. correndera*.

The PCA analysis showed subspecies *A. c. correndera* and *A. c. catamarcae* were clearly differentiated from the other groups (Figs. 4A–4C). The subspecies *A. c. correndera* was more differentiated along PC3, associated with climatic variables (bio3 and bio7) related with temperature oscillations (e.g., Isothermality and Temperature Annual Range, respectively), whereas the subspecies *A. c. catamarcae* was more differentiated along PC1, associated with precipitation variables (bio12 and bio14) (see Table S2 for relationships between PCs and climatic variables). Given that subspecific entities were not supported by genetic data, we rather focused on analyzing niche divergence patterns between the two mtDNA lineages (clade A and B, Fig. 2), that may correspond to incipient species. When analyzing differences between lineages from the mtDNA tree, we found significant differences along all three climatic axes (Table S4). However, these differences are not surprising given that these lineages are allopatric, and environmental differences are expected to increase with geographic distance in almost any system. Comparing these differences to null models of background divergence, we found evidence of niche differentiation for the climatic dimension associated with precipitations (PC1, *p* = 0.004), whereas the dimension associated with thermal oscillations (PC3) showed evidence of niche conservatism, with climatic distances between sites lower than expected under the null model (*p* = 0.014) (Fig. S5).

## Discussion

### Phylogeographic pattern and divergence times

Genealogy supports two main lineages within the *Anthus correndera* complex, one in the Andean Altiplano (clade A) and another in the southern South American lowlands (clade B). However, the monophyly of B is poorly supported and did not show a structured pattern of geographic differentiation congruent with current morphology-based taxonomy of *A. correndera*. We found *A. antarcticus* to be embedded within the lowlands clade, and unlike what was reported by Voelker (1999) and Van Els & Norambuena (2018) in their analyses of the *Anthus* genera, this species seems not closer to *correndera* or *grayi* than to *chilensis*-*catamarcae*. Unfortunately, the low sample sizes of some of the subspecies/regions could result in the low resolution of clade B. According to our results, the first divergence between highland and lowland taxa occurred between the end of the Mindel/Kansas glacial period and the beginning of an interglacial period. Our times of origin for *A. correndera* indicate a much more recent origin than previous estimates by Voelker (1999), due to which he inferred the origin of the *A. correndera*–*A. antarcticus* complex to be around 1.0 Mya, during the Pleistocene. However, given the low support of B, the divergence date with respect to A must be considered with caution. The inclusion of more samples and two methods of calibration in our analysis improved the divergence time estimation in relation to Voelker (1999).

### Niche divergence in climatic variables

Beyond the difference in altitude between clades A and B, which in some areas of northern Chile and Argentina may vary about 1,000 to 1,300 m, from sea level to Altiplano, there were clear differences in climatic conditions. However, climatic differences between non-overlapping areas can be expected due to spatial autocorrelation and not necessarily due to niche divergence (Costa et al., 2008). This is why we tested for niche divergence using a background test that contrasts levels of observed differences against those expected under a null model (only autocorrelation). Strikingly, we found evidence of niche divergence for variables associated with precipitation. These variables can be important drivers of speciation as precipitation can strongly impact food availability and areas of foraging both in time and space (Grant & Grant, 2002), which in turn may cause reproductive isolation due to shifts in temporal and spatial mating behavior (Nosil, 2012). Surprisingly, the background test also revealed that despite strong climatic differences between the Altiplano and the lowland areas used by *A. correndera*’s clades A and B, these incipient lineages tended to use sites that were more similar than expected by autocorrelation for variables associated with thermal oscillations. This can be interpreted as evidence of niche conservatism and suggests that thermal oscillations could be an important aspect for the fitness of this species complex.

Differentiation driven by niche divergence can be augmented by trans-glacial periods, geological characteristics of the area, and changing hydrology that can generate further fragmentation of grasslands, reducing the connectivity between populations (Cosacov et al., 2010). This is also supported by the ENM results, which suggest a pattern of low historical connectivity between lowlands and highlands (see Fig. 3B). However our gene flow analyses suggest the presence of migration mainly from lowlands to highlands. The poor support of some branches within clade B may be a result of rapid diversification during the middle Pleistocene and the dynamics of their habitat during this period (Sanín et al., 2009). This is common in species whose diversification occurred during the Pleistocene (Pavlova et al., 2005; Sanín et al., 2009; Van Els, Cicero & Klicka, 2012; Lougheed et al., 2013; Campagna et al., 2014; Li et al., 2016). Despite the clear difference between clade A and B, the low genetic differentiation within clade B suggest high levels of gene flow or incomplete lineage sorting. However, this also could be due to the small portion of the genome that we used (*cf.* Funk & Omland, 2003). These alternatives cannot be reliably separated with the present data. ENM gives some signs of possible connections between grasslands that could favor connectivity, mainly of lowlands populations in some refugial areas during the LGM (see Fig. 3B). Some areas like the Bolivian—Argentinian Altiplano and Eastern Patagonia (Fig. 3B) probably favored gene flow between populations and generated loss of incipient divergence (Huang & Knowles, 2015). For other Andean Altiplano vertebrate taxa, barriers such as rivers, lakes and salt flats, played an important role in shaping their genetic differentiation (e.g., Victoriano et al., 2015; Rivera et al., 2016), but phylogeographic structure is low even in non-vagile species (Correa et al., 2010; Vila et al., 2013; Victoriano et al., 2015). So, considering the high vagility of *A. correndera,* we expected little phylogeographic structure in areas that likely had high connectivity. For example, in the case of the Andean Altiplano, within the distribution of clade A, some basins between Chile and Bolivia probably permitted a historical connection of populations (Victoriano et al., 2015) that even could have existed until some centuries ago, favored by drastic climatic variations in this area (Latorre et al., 2003; Rech et al., 2003; Valero-Garcés et al., 2003). The connectivity between suitable habitats for *A. correndera* was likely maintained during postglacial periods, when melting ice raised the water level of lakes, extending their coverage (Ochsenius, 1986) and probably flooding or saturating large areas that currently do not form water bodies or wetlands (Victoriano et al., 2015). For the lowland populations, especially in Patagonia, a similar pattern of high connectivity could be favored by the glacial distribution that left some stable areas (identified in the ENM analysis), which were concordant with the lowland glacial refuges identified by Sérsic et al. (2011). These areas that conserved high genetic diversity, such as the West-Chilean coast and northern Neuquén, could have played a role in post-glacial colonization (Sérsic et al., 2011).

Based on geographic proximity, it is logical to propose that the South Georgia resident *A. antarcticus* probably originated from ancestors that colonized the island group from MFI. However, our analysis did not recover this demographic history. As shown by habitat dynamics (see Fig. 3) and the association of continental haplotypes with MFI, such relationship is not evident. The phylogenetic uncertainty could be caused by incomplete lineage sorting and by the small portion of the genome that we sampled. A multilocus analysis of the systematics of Neotropical *Anthus* recovered a close relationship between *grayi* and *antarcticus* (Van Els & Norambuena, 2018). So, the colonization of South Georgia by the ancestor of *A. antarcticus* could be explained by one or multiple founder events from continent to both islands facilitated by the low distance between these areas and the continent during the glacial periods. Campagna et al. (2012b) suggested that the poor genetic differentiation between continental (*A. c. chilensis*) and MFI populations (*A. c. grayi*) could be explained by different levels of migration or multiple colonization events (mainland-island flow), an idea supported by our ENM results, which suggest an increase in connectivity between the continent and MFI during the LGM. However, the timing and mode of this process require further testing with more data (e.g., multilocus or genome-wide), more samples (from islands), and model-based phylogeographic analyses.

### Historical demography and ENM

The ENM results suggest that suitable habitat for *A. correndera* was less extensive in the LGM relative to the present. This is contradictory to the general idea that grasslands increased their extension during the LGM (Haffer, 1969). An increase of grasslands was only evident in the Amazon Basin and the northern Andes (Salgado-Labouriau, 1991; Van Der Hammen, 1979; Markgraf, 1993; Webb, 1978) but our results suggest that in southern South America the process apparently was different, with a slight reduction or displacement of grassland habitat. This idea is also supported by studies of other Patagonian endemic taxa such as lizards (Breitman et al., 2012) and shrubs (Cosacov et al., 2013) that showed displacement of suitable habitat, rather than an increase in habitat during the LGM. These vegetational and climatic changes are explained by the latitudinal shifts and changes in intensity of the southern atmospheric circulation (Prieto, 1996). The lack of grassland expansion can explain why our historical demographic results suggest only modest increases in population size (Table 1 and Fig. S1). However, for the highland group the low sample size and their distribution could also be affecting these results (Heller, Chikhi & Siegismund, 2013; Grant, 2015). The possibility that some individuals of clade A are present in the *correndera* subspecies range was not detected due to low geographic coverage of this area. More analysis of the demographic history of the highland group is necessary. The discontinuities seem not to heavily impact patterns of genetic and lineage differentiation, at least within the widely distributed clade B. Nevertheless, there seems to be a historic association between the Andean Altiplano, that appears relatively isolated from southern areas, with the phylogeographic split between clades A and B.

In contrast to Patagonia, in the mountainous areas of the Andean Altiplano, it is expected that the connectivity among grasslands and their extent increased during the LGM, facilitating the connection of currently disconnected highland populations. In areas such as north-central Argentina migration between lowlands and the Andes could explain the presence of some individuals assigned to *catamarcae* that appear genetically closer to lowland individuals, suggesting introgression of mtDNA from lowlands to highlands. In some forested areas in the northern Andes, the advance of ice sheets and paramo during the LGM may have forced humid montane species into refugial areas (Ramírez-Barahona & Eguiarte, 2013). Alternatively, down-slope shifts of humid montane forest could have increased connectivity across lower elevation barriers, promoting dispersal and gene flow in humid-forest organisms (Vuilleumier, 1969; Graves, 1982; Benham et al., 2015). In this scenario, upslope shifts during interglacial periods promote divergence and changes in effective population sizes (Hooghiemstra, Wijninga & Cleef, 2006; Ramírez-Barahona & Eguiarte, 2013; Winger et al., 2015). A similar pattern can be expected for the central Andean Altiplano. In this area *A. c. calcaratus* and *A. c. catamarcae* have been distributed in a range where the refuges were smaller and more isolated during the LGM. Likely, each of these processes has played some role in different taxa and in different regions. Dispersal and vicariance likely work in conjunction in the Andes in a cyclical manner, with barriers becoming more permeable (facilitating dispersal) or impassable (facilitating vicariance) at different times, and it may not always be possible to separate these processes (Winger et al., 2015). In the particular case of *A. c. catamarcae* and *A. c. calcaratus*, it appears that some areas were more permeable to connections during the LGM, while others remained isolated.

## Conclusions

We obtained evidence of two main phylogenetic groups within *Anthus correndera*. Especially for the lowland group there are signs of incipient differentiation. The lack of congruence between morphology-based taxonomy and genetic differentiation in the *Anthus correndera* complex can be explained by a rapid colonization of grassland environments, which may have promoted a strong selection of certain morphological characters. However, a re-evaluation of morphological differences between subspecies is necessary, because the description of many of these was based on a low number of samples. Our results suggest that the grassland biome experienced distributional changes during the last glacial maximum, which affected the distribution of specialist grassland fauna. This distributional change was interrupted only by altitudinal differences that could influence the genetic population structure of a specialist species like *A. correndera*. Future analysis based on greater genomic coverage and the use of complementary information like vocalizations would be useful to complement these results.

##  Supplemental Information

10.7717/peerj.5886/supp-1 Figure S1 Locality sampling distributions for *Anthus correndera* complex used for the ENM analysis

10.7717/peerj.5886/supp-2 Figure S2 Species tree estimated with the *BEAST method Posterior probability values are above nodes. Two main clades are shown.

10.7717/peerj.5886/supp-3 Figure S3 Ultra-metric ND2 tree reconstructed with Bayesian inference in BEAST The values above the branches are the Posterior Probability, and below the branches are the age in Million Years (strict clock, 95% HPD).

10.7717/peerj.5886/supp-4 Figure S4 Bayesian skyline plots for the main clades A (highlands) and B (lowlands) of the *Anthus correndera* complex

10.7717/peerj.5886/supp-5 Figure S5 Background test for the niche differentiation of the *Anthus correndera* complex distribution

10.7717/peerj.5886/supp-6 Supplemental Information 1 Supplementary material Supplementary methods

10.7717/peerj.5886/supp-7 Table S1 Taxon sample list Taxon sample list, including institution (or ID), tissue number, country, region, locality and Genbank accession number per locus. Institution codes are as follows: AMNH, American Museum of Natural History; BAS, British Antarctic Survey; FIMNT, Falkland Islands Museum and National Trust; KU, University of Kansas Natural History Museum; LSUMZ, Louisiana State University Museum of Natural Science; UCCC, Universidad de Concepción; USNM, Smithsonian Institution National Museum of Natural History and UWBM, University of Washington Burke Museum.

10.7717/peerj.5886/supp-8 Table S2 Contributions of bioclim layers to the first three principal components (loadings) The table also includes a list of highly correlated bioclim layers that were removed.

10.7717/peerj.5886/supp-9 Table S3 Samples of *Anthus correndera* and haplotype distributions for the ND2 mitochondrial gene

10.7717/peerj.5886/supp-10 Table S4 Results of the statistical test ( *t*-test) that examined for climatic differences between clades A and B

10.7717/peerj.5886/supp-11 Supplemental Information 2 ND2 sequences Anthus correndera complex

10.7717/peerj.5886/supp-12 Supplemental Information 3 Sequence data for MK092368 –MK092379

10.7717/peerj.5886/supp-13 Supplemental Information 4 Sequence data for MH781103 –MH781135

## Additional Information and Declarations

### DNA Deposition

The following information was supplied regarding the deposition of DNA sequences: GenBank accession numbers ND2: MH781103 –MH781135, ACOI9: MK092368 –MK092379; see Table S1. Sequence data can also be found in the Supplemental Information.

### Data Availability

The following information was supplied regarding data availability: The ND2 sequences are available in a Supplemental File (FASTA).
