# Supplementary figures and images for: First steps towards assessing the evolutionary history and phylogeography of a widely distributed Neotropical grassland bird (Motacillidae: Anthus correndera)
Source: PeerJ. 2018 Nov 21;6:e5886. doi: 10.7717/peerj.5886 (PMC6252069; doi:10.7717/peerj.5886)

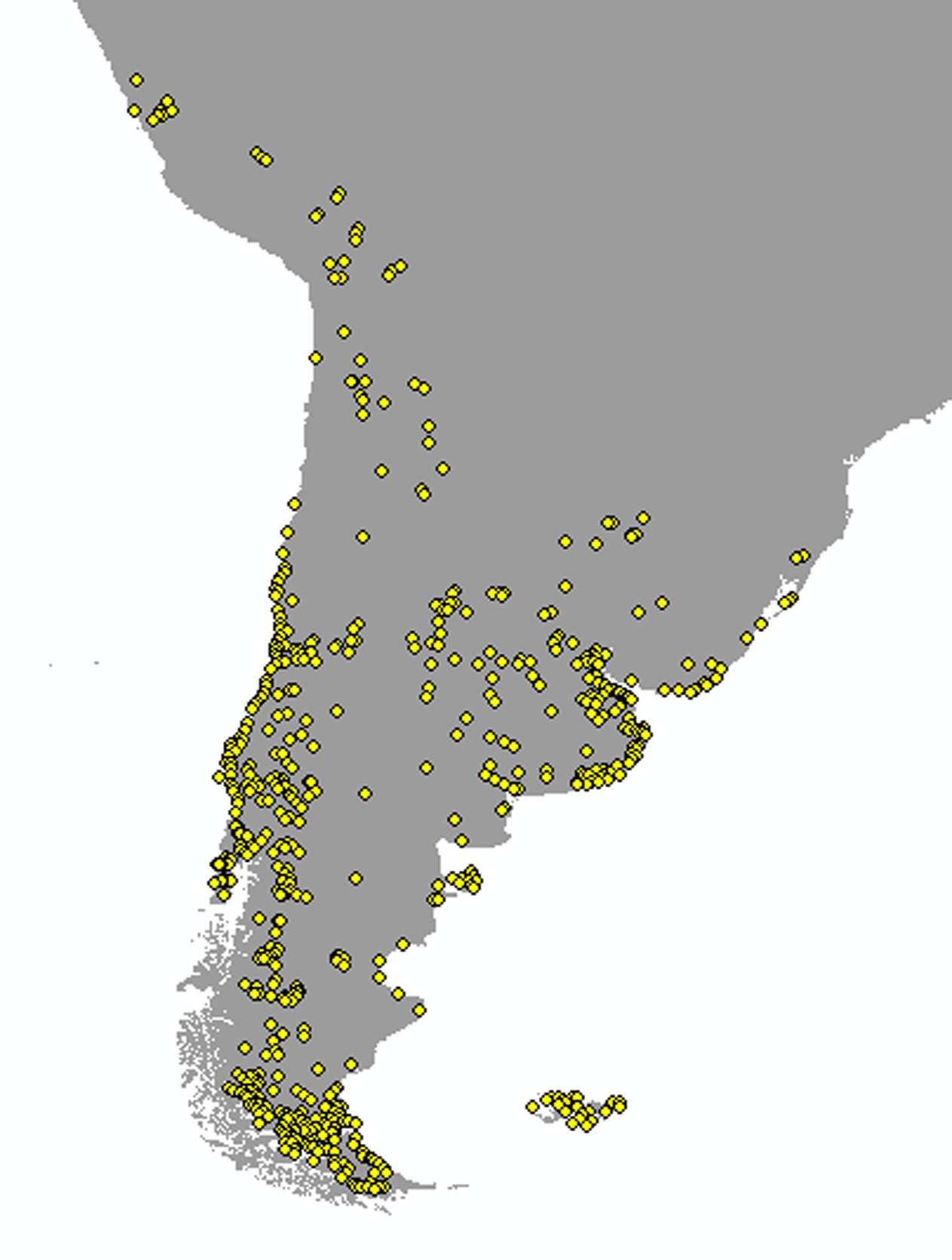

Supplement: Figure S1 [file peerj-06-5886-s001.png]

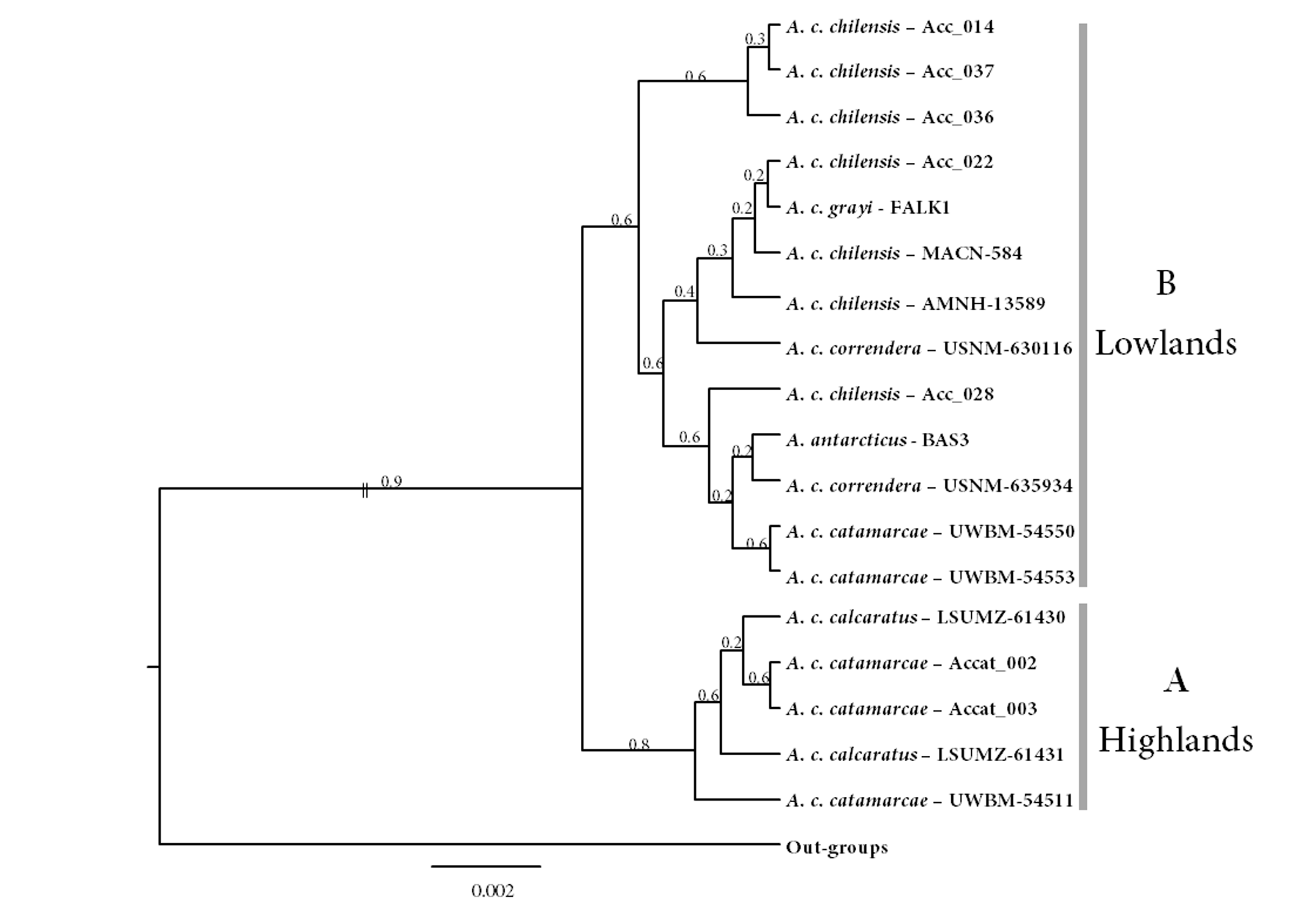

Supplement: Figure S2 — Posterior probability values are above nodes. Two main clades are shown. [file peerj-06-5886-s002.png]

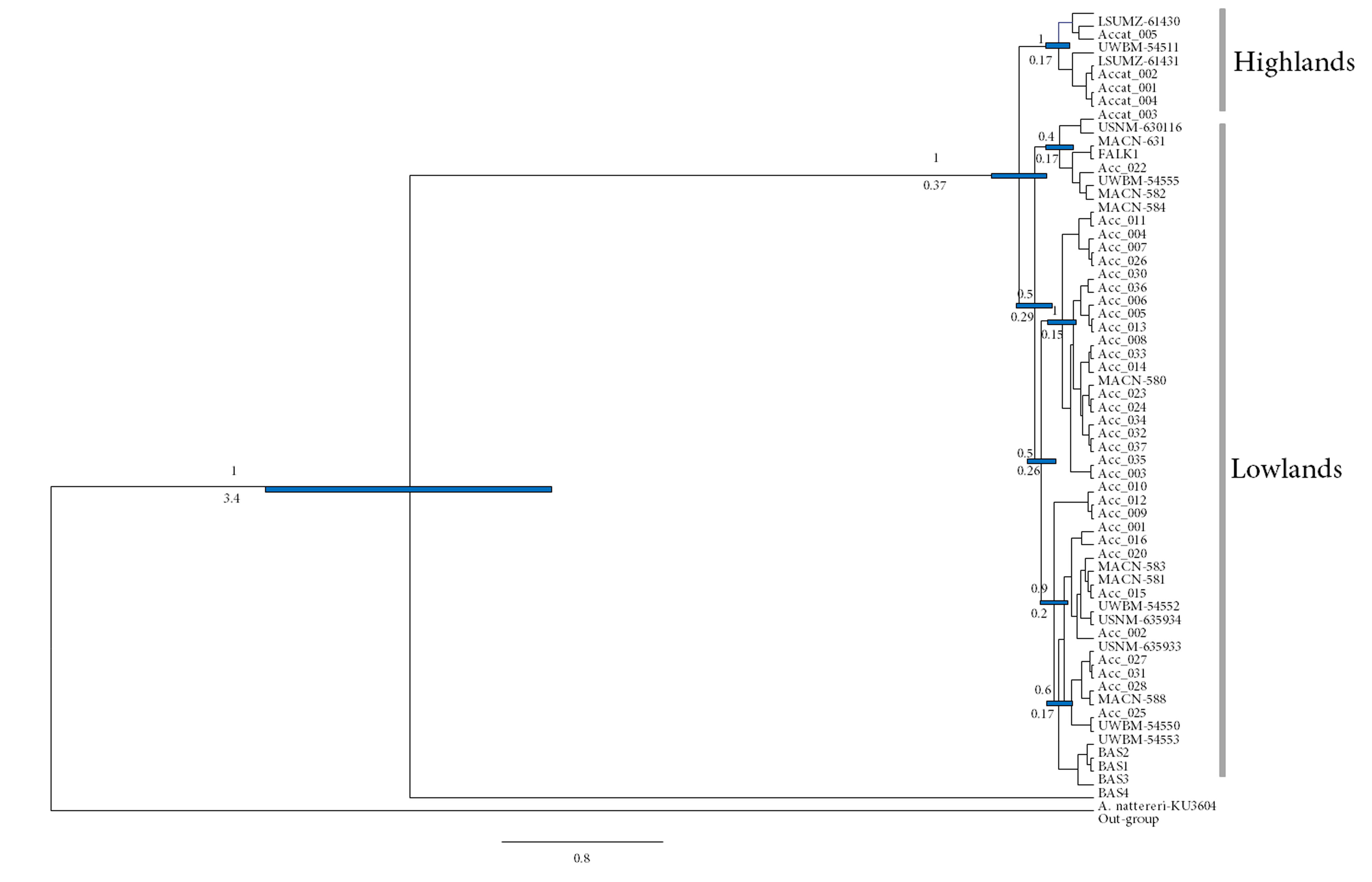

Supplement: Figure S3 — The values above the branches are the Posterior Probability, and below the branches are the age in Million Years (strict clock, 95% HPD). [file peerj-06-5886-s003.png]

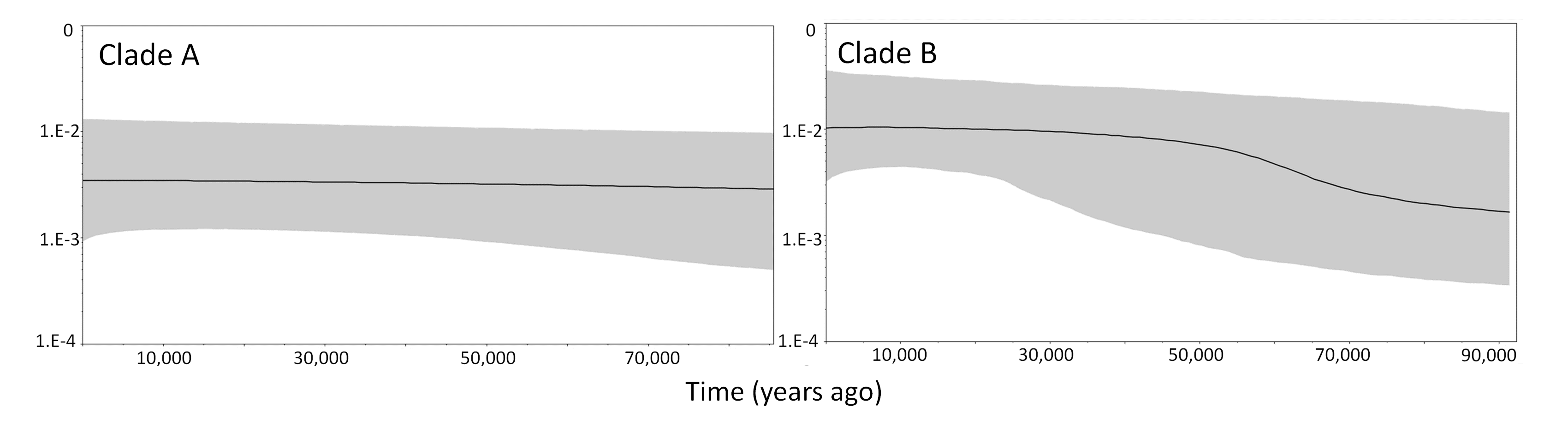

Supplement: Figure S4 [file peerj-06-5886-s004.png]

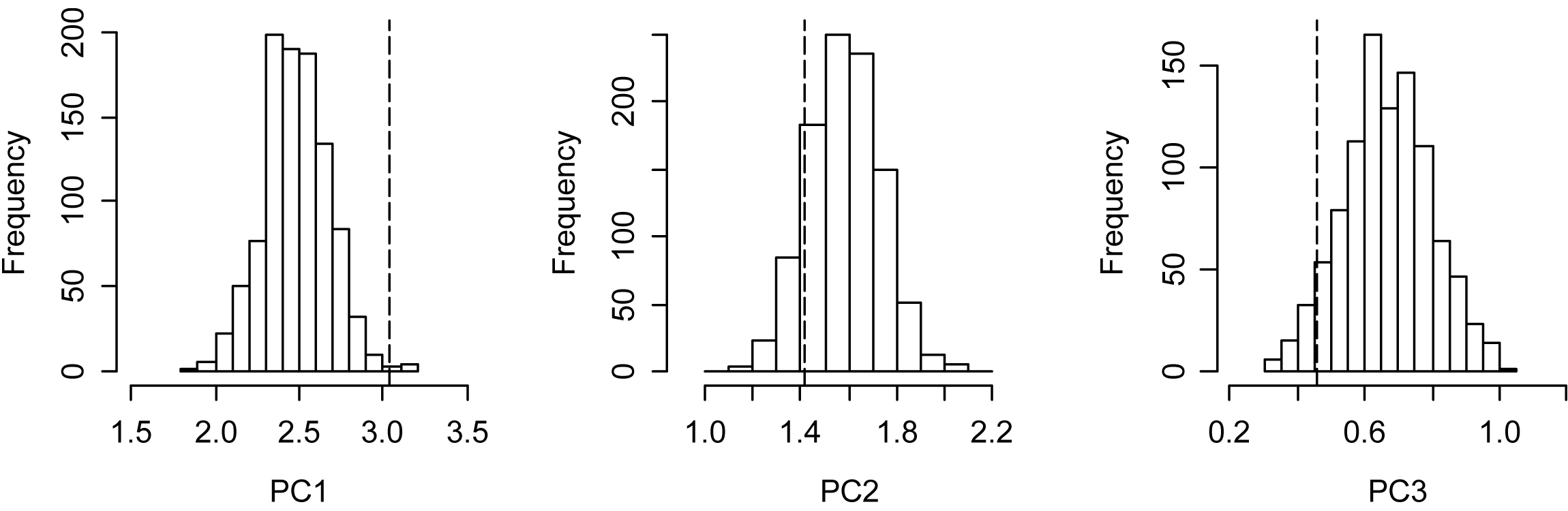

Supplement: Figure S5 [file peerj-06-5886-s005.png]
