# Supplementary material for: First steps towards assessing the evolutionary history and phylogeography of a widely distributed Neotropical grassland bird (Motacillidae: Anthus correndera)
Source: PeerJ. 2018 Nov 21;6:e5886. doi: 10.7717/peerj.5886 (PMC6252069; doi:10.7717/peerj.5886)
Supplement: Supplemental Information 1 — Supplementary methods [file peerj-06-5886-s006.docx]

Supplementary material

*Methodological details*

Background test

background test were conducted in R (R Development Core Team, 2013)

The background test compares niche divergence between groups by comparing their differences in environmental space to a null distribution of environmental differences obtained from random background points.

1. Obtaining random background coordinate points. We first generated a polygon around the coordinate points for the groups to be compared. In this case, we compared clades A and B obtained from the phylogenetic analyses of *A. correndera* (see figure 1 and 2 in the main text). The polygons were built using the **Sample by Buffered MCP** tool implemented in the SDMtoolbox package (Brown 2014) for ArcGIS 10.2 (ESRI Inc.). This tool generates a raster that can be easily converted to a polygon feature in ArcGIS. Consequently, each polygon was loaded into R to generate 1000 random points with the *spsample* R-function. Environmental data was obtained by using the R-package *raster* which has tools to extract data from the bioclimatic layers for each coordinate point.
2. PCA analyses. PCA analyses were conducted to create environmental axes from which niche differences can be assessed between groups. PCA analyses were conducted using the environmental analyses for both empirical and random coordinate points using the R function *princomp()*, with the correlation matrix option. For analyses and also for graphical display, we retained the three first PC axes that explained 82% of the climatic variation for the empirical data (see Supplementary Table S2).
3. Testing niche divergence: PC scores were used to assess mean differences between areas of distribution of clade A and B. Observed climatic differences between these clades were calculate for each of the three climatic axes that explained the highest amount of variation (PC1-3) by using the mean Euclidean distance. Then, for each climatic axis, we calculated distances between areas using the random background points, sampling a proportion of 10% of the points each time. We repeated this step 1000 times to generate a Null distribution of distances. Evidence of niche divergence is obtained when the observed mean climatic distance is equal or higher than the 95% of the distances in the Null distribution. Similarly, evidence for niche conservatism is obtained when the mean climatic distance is equal or smaller than the 95% of the distances in the Null distribution. Niche divergence is only valid if empirical differences are also statistically significant (under t-tests).

References

Brown JL (2014) SDMtoolbox: A python-based GIS toolkit for landscape genetic, biogeographic and species distribution model analyses. Methods Ecol Evol 5:694–700. doi: 10.1111/2041-210X.12200

McCormack JE, Zellmer AJ, Knowles LL (2010) Does niche divergence accompany allopatric divergence in Aphelocoma jays as predicted under ecological speciation? Insights from tests with niche models. Evol Int J Org Evol 64:1231–1244.
