# Supplementary material for: First steps towards assessing the evolutionary history and phylogeography of a widely distributed Neotropical grassland bird (Motacillidae: Anthus correndera)
Source: PeerJ. 2018 Nov 21;6:e5886. doi: 10.7717/peerj.5886 (PMC6252069; doi:10.7717/peerj.5886)
Supplement: Table S1 — Taxon sample list, including institution (or ID), tissue number, country, region, locality and Genbank accession number per locus. Institution codes are as follows: AMNH, American Museum of Natural History; BAS, British Antarctic Survey; FIMNT, Falkland Islands Museum and National Trust; KU, University of Kansas Natural History Museum; LSUMZ, Louisiana State University Museum of Natural Science; UCCC, Universidad de Concepción; USNM, Smithsonian Institution National Museum of Natural History and UWBM, University of Washington Burke Museum. [file peerj-06-5886-s007.docx]

Table S1

Taxon sample list, including institution (or ID), tissue number, country, region, locality (abbreviation) and GenBank accession number per locus. Institution codes are as follows: AMNH, American Museum of Natural History; BAS, British Antarctic Survey; FIMNT, Falkland Islands Museum and National Trust; KU, University of Kansas Natural History Museum; LSUMZ, Louisiana State University Museum of Natural Science; UCCC, Universidad de Concepción; USNM, Smithsonian Institution National Museum of Natural History and UWBM, University of Washington Burke Museum.

| **Nº** | **ID/Sample** | **Country** | **Region/District** | **Locality** | **Taxonomy** | **ND2** | **ACOI9** |
| --- | --- | --- | --- | --- | --- | --- | --- |
| 1 | Acc_001 | Chile | Biobío | Andalién (AN) | *A. c. chilensis* | MH781103 | - |
| 2 | Acc_003 | Chile | Biobío | Andalién (AN) | *A. c. chilensis* | MH781104 | - |
| 3 | Acc_004 | Chile | Biobío | Andalién (AN) | *A. c. chilensis* | MH781105 | - |
| 4 | Acc_005 | Chile | Biobío | Andalién (AN) | *A. c. chilensis* | MH781106 | - |
| 5 | Acc_006 | Chile | Biobío | Andalién (AN) | *A. c. chilensis* | MH781107 | - |
| 6 | Acc_007 | Chile | Biobío | Andalién (AN) | *A. c. chilensis* | MH781108 | - |
| 7 | Acc_008 | Chile | Biobío | Andalién (AN) | *A. c. chilensis* | MH781109 | - |
| 8 | Acc_009 | Chile | Biobío | Arauco (AR) | *A. c. chilensis* | MH781110 | - |
| 9 | Acc_010 | Chile | Biobío | Arauco (AR) | *A. c. chilensis* | MH781111 | - |
| 10 | Acc_011 | Chile | Biobío | Arauco (AR) | *A. c. chilensis* | MH781112 | - |
| 11 | Acc_012 | Chile | Biobío | Arauco (AR) | *A. c. chilensis* | MH781113 | - |
| 12 | Acc_013 | Chile | Biobío | Arauco (AR) | *A. c. chilensis* | MH781114 | - |
| 13 | Acc_014 | Chile | Biobío | Arauco (AR) | *A. c. chilensis* | MH781115 | MK092368 |
| 14 | Acc_015 | Chile | Araucanía | Lonquimay (LO) | *A. c. chilensis* | MH781116 | - |
| 15 | Acc_016 | Chile | Araucanía | Lonquimay (LO) | *A. c. chilensis* | MH781117 | - |
| 16 | Acc_018 | Chile | Maule | Putú (PU) | *A. c. chilensis* | MH781118 | - |
| 17 | Acc_019 | Chile | Maule | Putú (PU) | *A. c. chilensis* | MH781119 | - |
| 18 | Acc_020 | Chile | Maule | Putú (PU) | *A. c. chilensis* | MH781120 | - |
| 19 | Acc_022 | Chile | Aysén | Coyhaique Alto (CA) | *A. c. chilensis* | MH781121 | MK092369 |
| 20 | Acc_023 | Chile | Aysén | Balmaceda (BA) | *A. c. chilensis* | MH781122 | - |
| 21 | Acc_024 | Chile | Aysén | Balmaceda (BA) | *A. c. chilensis* | MH781123 | - |
| 22 | Acc_025 | Chile | Aysén | Balmaceda (BA) | *A. c. chilensis* | MH781124 | - |
| 23 | Acc_026 | Chile | Magallanes | Pto. Natales (PN) | *A. c. chilensis* | MH781125 | - |
| 24 | Acc_027 | Chile | Magallanes | Pto. Natales (PN) | *A. c. chilensis* | MH781126 | - |
| 25 | Acc_028 | Chile | Magallanes | Pta. Arenas (PA) | *A. c. chilensis* | MH781127 | MK092370 |
| 26 | Acc_030 | Chile | Magallanes | Pta. Arenas (PA) | *A. c. chilensis* | MH781128 | - |
| 27 | Acc_031 | Chile | Magallanes | Pta. Arenas (PA) | *A. c. chilensis* | MH781129 | - |
| 28 | Acc_032 | Chile | Coquimbo | Huentelauquén (HU) | *A. c. chilensis* | MH781130 | - |
| 29 | Acc_033 | Chile | Coquimbo | Huentelauquén (HU) | *A. c. chilensis* | MH781131 | - |
| 30 | Acc_034 | Chile | Coquimbo | Huentelauquén (HU) | *A. c. chilensis* | MH781132 | - |
| 31 | Acc_035 | Chile | Coquimbo | Huentelauquén (HU) | *A. c. chilensis* | MH781133 | - |
| 32 | Acc_036 | Chile | Coquimbo | Huentelauquén (HU) | *A. c. chilensis* | MH781134 | MK092371 |
| 33 | Acc_037 | Chile | Coquimbo | Huentelauquén (HU) | *A. c. chilensis* | MH781135 | MK092372 |
| 34 | MACN-580 | Argentina | Rio Negro | Pilcaniyeu (PI) | *A. c. chilensis* | MK121690 | - |
| 35 | MACN-581 | Argentina | Rio Negro | Pilcaniyeu (PI) | *A. c. chilensis* | MK121691 | - |
| 36 | MACN-582 | Argentina | Rio Negro | Pilcaniyeu (PI) | *A. c. chilensis* | MK121692 | - |
| 37 | MACN-583 | Argentina | Rio Negro | Pilcaniyeu (PI) | *A. c. chilensis* | MK121693 | - |
| 38 | MACN-584 | Argentina | Rio Negro | Pilcaniyeu (PI) | *A. c. chilensis* | MK121694 | MK092373 |
| 39 | MACN-588 | Argentina | Rio Negro | Pilcaniyeu (PI) | *A. c. chilensis* | MK121695 | - |
| 40 | MACN-631 | Argentina | Rio Negro | Pilcaniyeu (PI) | *A. c. chilensis* | MK121696 | - |
| 41 | Accat_001 | Chile | Antofagasta | Chiu-Chiu (CH) | *A. c. catamarcae* | MK121697 | - |
| 42 | Accat_002 | Chile | Antofagasta | Chiu-Chiu (CH) | *A. c. catamarcae* | MK121698 | MK092377 |
| 43 | Accat_003 | Chile | Antofagasta | Chiu-Chiu (CH) | *A. c. catamarcae* | MK121699 | MK092378 |
| 44 | Accat_004 | Chile | Antofagasta | Vados de Putana (VP) | *A. c. catamarcae* | MK121700 | - |
| 45 | Accat_005 | Chile | Antofagasta | Vados de Putana (VP) | *A. c. catamarcae* | MK121701 | - |
| 46 | UWBM-54511 | Argentina | Tucuman | San Miguel de Tucuman (SM) | *A. c. catamarcae* | MF320001 | MF320044 |
| 47 | UWBM-54550 | Argentina | Tucuman | San Miguel de Tucuman (SM) | *A. c. catamarcae* | MK121702 | MK092375 |
| 48 | UWBM-54551 | Argentina | Tucuman | San Miguel de Tucuman (SM) | *A. c. catamarcae* | MK121706 | - |
| 49 | UWBM-54552 | Argentina | Tucuman | San Miguel de Tucuman (SM) | *A. c. catamarcae* | MK121703 | - |
| 50 | UWBM-54553 | Argentina | Tucuman | San Miguel de Tucuman (SM) | *A. c. catamarcae* | MK121704 | MK092376 |
| 51 | UWBM-54555 | Argentina | Tucuman | San Miguel de Tucuman (SM) | *A. c. catamarcae* | MK121705 | - |
| 52 | USNM-630116 | Uruguay | Tacuarembó | Estancia La Rosada (TA) | *A. c. correndera* | MF319989 | MF320020 |
| 53 | USNM-635933 | Uruguay | Rocha | Estancia La Cañada (RO) | *A. c. correndera* | MK121708 | - |
| 54 | USNM-635934 | Uruguay | Rocha | Estancia La Cañada (RO) | *A. c. correndera* | MK121709 | MK092374 |
| 55 | LSUMZ-61430 | Perú | Puno | Puno (PUN) | *A. c. calcaratus* | MF319985 | MF320016 |
| 56 | LSUMZ-61431 | Perú | Puno | Puno (PUN) | *A. c. calcaratus* | MF319986 | MF320017 |
| 57 | FALK1 | FIMNT | Malvinas/Falklands | Malvinas/Falklands (MF) | *A. c. grayi* | MF320007 | MF320037 |
| 58 | BAS1 | - | South Georgia | South Georgia (SG) | *A. antarcticus* | MK121707 | - |
| 59 | BAS2 | - | South Georgia | South Georgia (SG) | *A. antarcticus* | MF320010 | MF320047 |
| 60 | BAS3 | - | South Georgia | South Georgia (SG) | *A. antarcticus* | MF320009 | MF320048 |
| 61 | BAS4 | - | South Georgia | South Georgia (SG) | *A. antarcticus* | - | MK092379 |
| 62 | KUSNM-116859 | Ecuador | Cotopaxi | Cotopaxi | *A. bogotensis bogotensis* | MF319979 | MF320027 |
| 63 | LSUMZ-431 | Peru | Piura | Piura | *A. bogotensis bogotensis* | MF320026 | MF320026 |
| 64 | KU-25127 | Peru | Ayacucho | Ayacucho | *A. bogotensis immaculatus* | MF320028 | MF320028 |
| 65 | AMNH-811977 | Venezuela | Mérida | Mérida | *A. bogotensis meridae* | MF320011 | - |
| 66 | AMNH-811978 | Venezuela | Mérida | Mérida | *A. bogotensis meridae* | MF320012 | - |
| 67 | USNM-645734 | Argentina | Tucumán | Tucumán | *A. bogotensis shiptoni* | MF320000 | MF320034 |
| 68 | UWBM-54394 | Argentina | Tucumán | Tucumán | *A. bogotensis shiptoni* | MF319999 | MF320033 |
| 69 | AMNH-797085 | Argentina | Córdoba | Córdoba | *A. chacoensis* | MF320008 | - |
| 70 | KU-21673 | Peru | Puno | Puno | *A. furcatus brevirostris* | MF319996 | MF320038 |
| 71 | KU-21681 | Peru | Puno | Puno | *A. furcatus brevirostris* | MF319997 | MF320039 |
| 72 | UWBM-54556 | Argentina | Tucumán | Tucumán | *A. furcatus furcatus* | MF347705 | MF320045 |
| 73 | USNM-635884 | Uruguay | Artigas | Artigas | *A. furcatus furcatus* | MF320002 | MF320046 |
| 74 | UWBM-54574 | Argentina | Corrientes | Corrientes | *A. hellmayri brasilianus* | MF319991 | MF320022 |
| 75 | USNM-630210 | Uruguay | Tacuarembo | Tacuarembo | *A. hellmayri brasilianus* | MF319990 | MF320021 |
| 76 | UCCC | Chile | Araucanía | Araucanía | *A. hellmayri dabbenei* | MF320013 | MF320049 |
| 77 | UCCC | Chile | Araucanía | Araucanía | *A. hellmayri dabbenei* | MF320014 | MF320050 |
| 78 | KU-9813 | Argentina | Jujuy | Jujuy | *A. hellmayri hellmayri* | MF319994 | MF320042 |
| 79 | UWBM-54528 | Argentina | Tucumán | Tucumán | *A. hellmayri hellmayri* | MF319995 | MF320043 |
| 80 | LSUMZ-44804 | Peru | La Libertad | La Libertad | *A. lutescens peruvianus* | MF319984 | MF320032 |
| 81 | LSUMZ-48218 | Peru | Lima | Lima | *A. lutescens peruvianus* | MF319983 | MF320031 |
| 82 | KU-3604 | Paraguay | Itapua | Itapua | *A. nattereri* | MF319992 | MF320040 |
| 83 | KU-3665 | Paraguay | Itapua | Itapua | *A. nattereri* | MF319993 | MF320041 |
| 84 | LSUMZ-25702 | USA | North Dakota | North Dakota | *A. spraguei* | MF319980 | MF320023 |
| 85 | LSUMZ-21749 | USA | North Dakota | Louisiana | *A. spraguei* | MF319981 | MF320024 |
| 86 | UWBM-52816 | South Africa | Eastern Cape | Eastern Cape | *A. cinnamomeus* | AY329410 | - |
| 87 | UWBM-75556 | Russia | Primorsky Krai | Primorsky Krai | *A. gustavi* | HM538396 | - |
| 88 | LSU-53141 | USA | California | California | *A. rubescens* | MF320015 | - |
| 89 | FMNH-358350 | Philippines | Sibuyan | Sibuyan | *A. rufulus* | KP671566 | - |
