# Supplementary material for: First steps towards assessing the evolutionary history and phylogeography of a widely distributed Neotropical grassland bird (Motacillidae: Anthus correndera)
Source: PeerJ. 2018 Nov 21;6:e5886. doi: 10.7717/peerj.5886 (PMC6252069; doi:10.7717/peerj.5886)
Supplement: Table S2 — The table also includes a list of highly correlated bioclim layers that were removed. [file peerj-06-5886-s008.docx]

Table S2. Contributions of bioclim layers to the first three principal components (loadings). The table also includes a list of highly correlated bioclim layers that were removed.

| Bioclim codes | PC1 | PC2 | PC3 | Bioclim layer description | Other bioclim layers highly correlated to (pearson>0.8) |
| --- | --- | --- | --- | --- | --- |
| bio1 | 0.06 | -0.38 | -0.35 | Annual Mean Temperature | bio9(0.92); bio8(0.94); bio6(0.96); bio5(0.9); bio10(0.95); bio11(0.98) |
| bio2 | -0.31 | **-0.47** | -0.18 | Mean Diurnal Range (Mean of monthly (max temp - min temp)) | None |
| bio3 | -0.27 | -0.29 | **0.47** | Isothermality (BIO2/BIO7) (* 100) | bio4(-0.9) |
| bio7 | -0.19 | -0.37 | **-0.55** | Temperature Annual Range (BIO5-BIO6) | bio4(0.87) |
| bio12 | **0.43** | -0.34 | 0.19 | Annual Precipitation | bio13(0.90); bio16(0.92) |
| bio14 | **0.51** | -0.10 | -0.09 | Precipitation of Driest Month | bio17(0.99) |
| bio15 | -0.31 | -0.38 | 0.36 | Precipitation Seasonality (Coefficient of Variation) | None |
| bio18 | 0.39 | -0.20 | -0.14 | Precipitation of Warmest Quarter | None |
| bio19 | 0.32 | -0.33 | 0.36 | Precipitation of Coldest Quarter | None |
