# Supplementary material for: First steps towards assessing the evolutionary history and phylogeography of a widely distributed Neotropical grassland bird (Motacillidae: Anthus correndera)
Source: PeerJ. 2018 Nov 21;6:e5886. doi: 10.7717/peerj.5886 (PMC6252069; doi:10.7717/peerj.5886)
Supplement: Table S3 [file peerj-06-5886-s009.docx]

Table S3.

Samples of *Anthus correndera* and haplotype distributions for the ND2 mitochondrial gene.

| Haplotype | Nº of sequences | Samples |
| --- | --- | --- |
| Hap_1 | 1 | Acc_020 |
| Hap_2 | 1 | Acc_016 |
| Hap_3 | 8 | Acc_002; Acc_015; MACN-581; MACN-583; USNM- 635934; UWBM-54550; UWBM-54552; UWBM-54553 |
| Hap_4 | 3 | Acc_022; MACN-584; FALK1 |
| Hap_5 | 12 | Acc_023; Acc_024; Acc_032; Acc_033; Acc_034; Acc_035; Acc_037; Acc_004; Acc_006; Acc_011; Acc_014; MACN-580 |
| Hap_6 | 2 | Acc_025; MACN-588 |
| Hap_7 | 3 | Acc_026; Acc_030; Acc_007 |
| Hap_8 | 3 | Acc_027; Acc_028; Acc_031 |
| Hap_9 | 1 | Acc_036 |
| Hap_10 | 3 | Acc_001; Acc_009; Acc_012 |
| Hap_11 | 2 | Acc_003; Acc_010 |
| Hap_12 | 3 | Acc_005; Acc_008; Acc_013 |
| Hap_13 | 6 | Accat_001; Accat_002; Accat_003; Accat_004; Accat_005; LSUMZ-61430 |
| Hap_14 | 1 | MACN-582 |
| Hap_15 | 1 | MACN-631 |
| Hap_16 | 1 | USNM-635933 |
| Hap_17 | 1 | UWBM-54555 |
| Hap_18 | 1 | LSUMZ-61431 |
| Hap_19 | 1 | USNM-630116 |
| Hap_20 | 1 | UWBM-5451 |
| Hap_21 | 4 | BAS1; BAS2; BAS3; BAS4 |
