# Supplementary material for: First steps towards assessing the evolutionary history and phylogeography of a widely distributed Neotropical grassland bird (Motacillidae: Anthus correndera)
Source: PeerJ. 2018 Nov 21;6:e5886. doi: 10.7717/peerj.5886 (PMC6252069; doi:10.7717/peerj.5886)
Supplement: Table S4 [file peerj-06-5886-s010.docx]

Table S4.

Results of the statistical test (t-test) that examined for climatic differences between clades A and B.

| Climatic axis | t | degree of freedom | p-value |
| --- | --- | --- | --- |
| PC1 | -17.83 | 65.844 | 2.20E-16 |
| PC2 | -8.396 | 50.52 | 3.77E-11 |
| PC3 | 2.873 | 57.33 | 0.005692 |
